# Follicular fluid steroid and gonadotropic hormone levels and mitochondrial function from exosomes predict embryonic development

**DOI:** 10.3389/fendo.2022.1025523

**Published:** 2022-11-09

**Authors:** Li Yu, Miao Liu, Shiji Xu, Zhenxin Wang, Te Liu, Jiaye Zhou, Doudou Zhang, Xi Dong, Baishen Pan, Beili Wang, Suying Liu, Wei Guo

**Affiliations:** ^1^ Department of Laboratory Medicine, Zhongshan Hospital, Fudan University, Shanghai, China; ^2^ Reproductive Medicine Center, Zhongshan Hospital, Fudan University, Shanghai, China; ^3^ Shanghai Geriatric Institute of Chinese Medicine, Shanghai University of Traditional Chinese Medicine, Shanghai, China; ^4^ Department of Laboratory Medicine, Xiamen Branch, Zhongshan Hospital, Fudan University, Xiamen, China; ^5^ Department of Laboratory Medicine, Wusong Branch, Zhongshan Hospital, Fudan University, Shanghai, China

**Keywords:** LH, FSH, follicular fluid, mitochondrial abnormalities, oocyte competence

## Abstract

**Purpose:**

Human follicular fluid (FF) is a complex biological fluid that contributes to the micro-environment of oocyte development. The aim of this study was to evaluate the role of steroid and gonadotropic hormones levels and mitochondrial function in embryo development during *in vitro* fertilization cycles.

**Methods:**

This was a cohort study of 138 women receiving IVF/ICSI, including 136 FF samples from 109 infertile women. FF steroid and gonadotropic hormones levels were tested by liquid chromatography-tandem mass spectrometry (LC-MS/MS) and immunoassays. The mRNA expression levels of mitochondrial electron transport chain (ETC) complex genes from FF exosomes were detected by qPCR.

**Results:**

Analysis of these individual FF concentrations revealed that LH and FSH concentrations were higher in follicles in which the oocyte developed into a top quality (TQ) blastocyst (LH: 9.44 ± 2.32mIU/ml, FSH: 9.32 ± 1.01mIU/ml) than those in which there was a failure of fertilization (LH: 5.30 ± 0.84mIU/ml, FSH: 6.91 ± 0.62mIU/ml). In contrast, follicular cortisone concentrations were lower for oocytes that resulted in a TQ blastocyst (12.20 ± 0.82mIU/ml). The receiver operating characteristic analysis showed that FF LH and FSH levels predicted TQ blastocyst with excellent AUC value of 0.711 and 0.747. Mitochondrial ETC complex I and III mRNA levels were increased in the FF exosomes of TQ blastocyst. Correlation analysis showed that mRNA levels of ETC complex I was positively correlated with LH and FSH levels in FF.

**Conclusion:**

The levels of FF steroid and gonadotropic hormones from single follicle can predetermine subsequent embryo development to some extent. Furthermore, impaired exosome mitochondrial dysfunction is a potiential event that causes hormone change in embryo development.

## Introduction

In the assisted reproductive technology (ART) cycle, only a few retrieved oocytes can develop into a fertilizable embryo (1). Despite multiple approaches of embryo selection such as morphological assessment, embryo imaging and preimplantation genetic testing, predetermine the quality and developmental potential of oocytes is still a major obstacle that IVF needs to overcome to increase pregnancy rates (2). In some cases doctors choose to transfer multiple embryos per treatment cycle to increase pregnancy rates which could increase the risk of complications for mother and offspring (3). As a result, further understanding the regulatory mechanisms of oocyte competence is important. The discovery of an accurate, non-invasive and low-cost test for predicting oocyte developmental potential may have a significant impact on assisted reproduction.

It is well known that the follicular fluid (FF) microenvironment can help to regulate oocyte maturation, oocyte quality and subsequent embryonic development (4). Previous studies have correlated follicular fluid steroid concentrations and embryonic outcomes and try to predict IVF outcomes (5, 6). In our study, we use a multi-analyte LC-MS/MS method (7) for 20 steroids and measured FF AMH, FSH, LH, PRL and hCG levels by immunoassays. The main objective of our study was to investigate the sex hormones in FF as potential biomarkers for successful development to a blastocyst stage embryo.

Exosomes are small membrane vesicles (30-200nm in diameter) secreted by a variety of living cells under normal or pathophysiological conditions (8). In ovarian follicles, bidirectional communication occurs between oocytes and somatic cells (granulosa cells and theca cells) in the follicular fluid and exosomes are one of their communication carriers. In several previous studies of human follicular fluid (9, 10), it has been clearly established that extracellular vesicle miRNAs secreted by ovarian follicular cells play an important role in follicular growth and oocyte maturation.

Completion of oocyte cytoplasmic and meiotic maturation is necessary for oocyte development. The competence of oocytes is the result of the combined action of multiple cellular processes. Mitochondria are organelles necessary for the production of energy (11). There is increasing evidence for their role in oocyte development and reproduction. In immature eggs, mitochondria are silent in transcription and bioenergy (12) and ATP production is slow (13). During oocyte maturation and early preimplantation embryo development, glycolysis and mitochondrial replication remain restricted until the blastocyst stage (14). MtDNA count does not change from metaphase II oocytes to blastocyst stage (15, 16). During preimplantation embryo development, mitochondria from the oocyte are the main source of ATP. Mitochondria also contribute in maintaining metabolic homeostasis. Alterations in mitochondrial functions are often related with peripheral insulin resistance and glucose intolerance (17, 18).

Exosomes containing mitochondrial proteins have been identified, and these new mitochondria derived EVs were called “mitovesicles”. D’Acunzo (19) demonstrated that brain-derived mitovesicles contain a specific subset of mitochondrial components whose levels and cargo were altered during pathophysiology. Mesenchymal stromal cells can package mitochondria for export into EVs, which may be a delivery strategy for cell-free mitochondria-targeted therapy (20). Studies on human FF exosomes and mitochondrial proteins are scarce. Our research identified mitochondrial mRNA in the FF exosomes and innovatively linked it to oocyte quality.

The aim of this prospective study was to investigate the possible relationship between follicle hormone levels and the developmental potential of oocytes to develop from fertilization into embryos. We also investigated the relationship between the expression of mitochondria ETC complexes and hormone metabolic involved in the intrafollicular environment of oocyte development.

## Materials and methods

### Subjects and inclusion/exclusion criteria

Samples were collected from January 2020 to May 2021. Written informed consent are obtained from the patients. The study was approved by the Ethical Committee of the Zhongshan Hospital, Fudan University (Shanghai, China), and conducted in compliance with the Population and Family Planning Law of the People’s Republic of China (The ethical approval number: B2021-665).

This study aims to evaluate the maternal contribution to embryo outcomes. All the recruited patients were under 38 years old and had a normal BMI range from 17.2 to 27.7 kg/m^2^. Patients recruited for this study were with normal ovulatory cycles. Patients also had normal baseline follicle stimulating hormone (FSH<25 U/L), basal antral follicle count (AFC>3), normal transvaginal ultrasonography and the presence of both normal ovaries. These infertile women were due to tubal factors, including hydrosalpinx and proximal tubal obstruction. These patients received the standard GnRH antagonist protocol as explained below. Women who may have adverse effects on oocyte or embryo quality and implantation such as PCOS and endometriosis and received pharmacological treatment for infertility in the past three months were excluded. Patients with genital tract infection, endometriosis, chromosomal abnormalities, myoma of uterus, diminished ovarian reserve, a cancer diagnosis and diabetes were also excluded. In addition, decreased fertilization rates and stunted blastocyst development due to male factors should be excluded (21). Therefore, the exclusion criteria were as follows: age is over 38 years; poor ovarian response to gonadotropin stimulation with less than 3 retrieved oocytes; moderate or severe male factor (based on semen quality on the day of oocyte retrieval).

### Clinical examination, stimulation protocol and oocyte maturation triggering

The patients’ BMI values, hormone levels, biochemical and ultrasound parameters were recorded. Patients were treated with a standard *in vitro* fertilization (IVF) antagonist stimulation protocol. Ovarian stimulation is standard GnRH antagonist protocol. Subcutaneous daily injections of 150-300 IU/day of recombinant FSH (Puregon, MSD, Courbevoie, France; Gonal-F, Merck-Serono, Lyon, France) or urinary FSH (hMG, Menotrophin for Injection, Livzon Pharmaceutical Group Inc, Guangdong, China) were started on the second day of the menstrual cycle. Gonadotropin doses will be determined based on individual patient’s characteristics. The initial dose of gonadotropin was individually selected according to age, body mass index, anti-Mullerian hormone (AMH), and antral follicle count (AFC). In our study, most patients use 150 IU/day of recombinant FSH as the initial dose. During controlled ovarian hyperstimulation, we monitored serum hormone levels, size and count of follicles, and endometrial thickness. The dose of gonadotropins was adjusted according to follicular development. GnRH antagonist (cetrorelix; Merck Serono, Darmstadt, Germany) is administered subcutaneously at a daily dose of 0.25mg when there is at least one follicle measuring≥12 mm in mean diameter on the trigger day. Final oocyte maturation will be triggered when more than two ovarian dominant follicles measuring ≥18 mm were visible by ultrasound. Final oocyte maturation will be achieved using either a single 250μg of recombinant hCG (rhCG, Ovitrelle, Serono, France) or 0.2mg injection of GnRH agonist (Triptoreline, Decapeptyl, Ipsen, France). Oocyte retrieval will be performed after 35-36h by transvaginal ultrasound-guided aspiration. The oocytes were retrieved in the same day of the cycle.

### Follicular fluid collection

Aspirate each follicle independently and collect the FF into a separate tube to match it with a single COC obtained from the same follicle. After the first ovarian puncture, remove the needle to flush and aspirate air until the tube is empty. The collected FFs were checked for erythrocytes; FFs with erythrocytes were excluded from the study. After oocyte isolation, FF was centrifuged at 14,000×g for 20min to remove cells and insoluble particles. The supernatant was then stored at −80°C for further analysis.

### Measurement of FF markers

20 steroids were measured in organic solvent extracts of 200 μl of follicular fluid samples using LC-MS/MS. Details of the LC-MS/MS methods are presented in the reference (7). Wang et al. verified the limit of detection, lower limit of quantification, specificity, matrix effect, accuracy and precision of LC-MS/MS assay. The coefficients of variation of the 20 analytes at the lower limit of quantification were all less than 15% (ranging from 1.84% to 14.96%). Specificity was evaluated according to the ratio of background peak area/analyte peak area. The background peak area/lower limit of quantification peak area was <15%. The intra-assay precision and inter-assay precision meet the condition of CV value <15%. LH, FSH, PRL, AMH, hCG are measured by immunoassays.

### Exosome isolation, RNA isolation and quantitative RT-PCR

The follicular fluid exosomes were isolated by ultracentrifugation. Transmission electron microscopy, nanoparticle tracking analysis and western blot analysis were used to characterize exosomes according to our previous study (22). Transmission electron microscopy, nanoparticle tracking detection were performed by Runan Medical Technology (Suzhou) Ltd., Co. According to the manufacturer’s instructions, total RNA was extracted by TRIzol (Invitrogen) and reverse- transcribed by SuperScript First-Strand cDNA System (Takara). Quantitative RT-PCR (qRT-PCR) was performed using the SYBR Green PCR master mix (Takara) and the StepOne Plus PCR system (Thermo Fisher Scientific). GAPDH was used as an endogenous control. Standard curves were normalized mRNA transcript levels that correspond to logarithmic values of input cDNA concentrations. The ΔCT-value was obtained by subtracting the GAPDH CT value from the CT value of for the genes of interest. The ΔCT of FF exosomes from no fertilisation after IVF group was used as the calibrator. Fold change was calculated according to the formula 2^−(ΔΔCT)^, where ΔΔCT was the difference between ΔCT and ΔCT calibrator value. Statistical significance was calculated between mRNA expression of FF exosomes in patients of no fertilisation after IVF and TQ blastocyst on the 5th day. The primer sequences are shown in **Table S1**.

### Statistical analysis

SPSS (Chicago, IL, USA) was used for statistical analysis. Comparison between two groups was performed using t-test for normally distributed variables and Mann-Whitney U test for non-normally distributed variables. When many groups were compared, Kruskal-Wallis one-way ANOVA was used. Pearson test was used for normally distributed variables, and Spearmen test was used for non-normally distributed parameters. The correlation between FF steroid hormones and other clinical and laboratory parameters was analyzed. Variables are expressed as mean and SEM. Statistical significance was defined as P < 0.05 for all comparisons.

## Results

### Study group and follicular fluid characteristics

**Figure 1:**
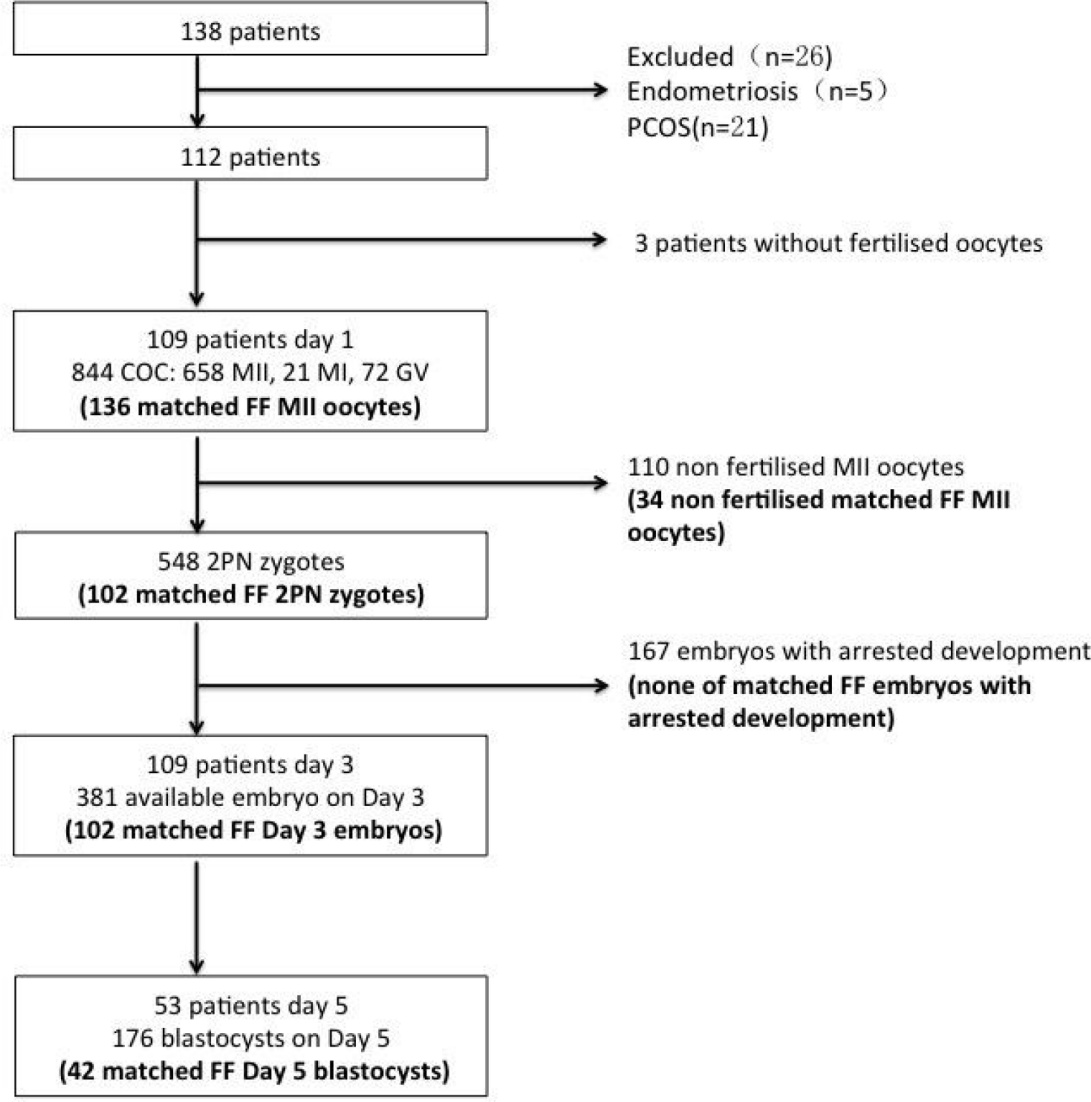
Flowchart of participants, oocytes, embryos and embryo transfer procedure in the study. FF—follicular fluid; COC—cumulus–oocyte complex, GV—germinal vesicle, MI immature metaphase I oocyte; MII—mature metaphase II oocyte; 2PN—2 pronuclear zygote; matched FF—embryo developed from an oocyte that has matched the FF sample in the study; TQ—top quality.

**Figure 2:**
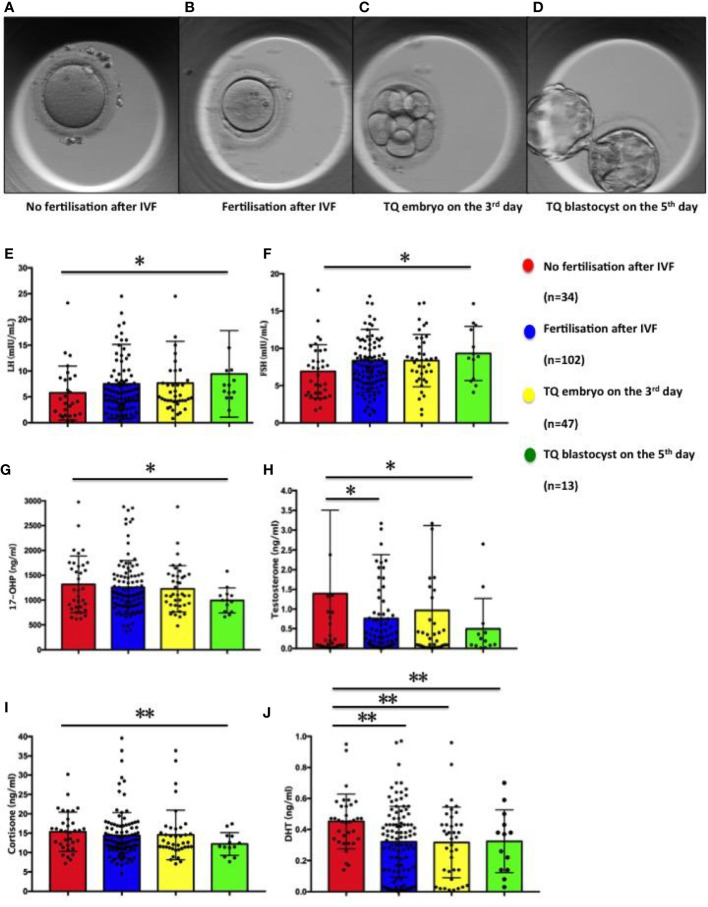
Figures of oocyte and embryo in different developmental states and distribution of steroid and gonadotropic hormone concentrations in follicular fluid in relation to the corresponding oocytes: fertilisation and subsequent embryo development. **(A)** Oocyte of no fertilisation after IVF; **(B)** Embryo fertilisation after IVF; **(C)** TQ embryo on the 3^rd^ day; **(D)** TQ blastocyst on the 5^th^ day; **(E)** LH; **(F)** FSH; **(G)** 17-hydroxy pregnenolone (17-OHP); **(H)** Testosterone; **(I)** Cortisone; **(J)** Dihydrotestosterone. Comparison between groups was performed using Kruskal-Wallis one-way ANOVA. *p< 0.05, **p< 0.01.

**Table 1** summarizes the characteristics of the patients who participated in the study. Of 138 patients enrolled between January 2020 and May 2021, 112 patients were enrolled. The embryonic development process of the oocytes matched with FF is shown in **Figure 1**. On the third day of culture, embryos with eight symmetrical, non-fragmented blastomeres were defined as high-quality embryos (**Figure 2C**). High-quality embryos will show the development of the inner cell mass (ICM), the appearance of the trophectoderm (TE) and the expansion of the blastocyst (**Figure 2D**) on the fifth day of the culture, at the blastocyst stage. Embryos underwent embryo assessment according to 2011 Istanbul consensus (23). Of the 136 oocytes with paired FF samples, 34 (25%) oocytes were not fertilized. The remaining 102 2PN embryos were cultured as single embryos on Day 3, while 42 embryos were cultured as single embryos on Day 5. The TQ embryo rate on the 3^rd^ day and 5^th^ day were 46.1% and 30.95% respectively.

**Table 1:** Patients and follicular fluid sample characteristics and embryological outcome of the oocytes with matched FF.

Patients clinical characteristics	
Age (year)	34.93 ± 0.481 (24-38)
BMI (kg/m^2^)	22.70 ± 0.33(17.2-27.7)
Basal serum LH (mIU/ml)	5.56 ± 0.24 (1.7-14.4)
Basal serum FSH (mIU/ml)	7.91 ± 0.30 (4.5-23.2)
Basal serum E2 (pmol/ml)	380.40 ± 89.87 (71.5-7019)
Basal serum P (nmol/l)	1.14 ± 0.45 (0.2-48.2)
Basal serum T (nmol/l)	0.86 ± 0.06 (0.2-4.7)
Basal serum PRL (mIU/l)	395.17 ± 20.56 (63.1-1126)
Number of oocytes retrieved	10.99 ± 0.75 (4-23)
Number of MII oocytes	8.49 ± 0.58 (3-20)
**Follicular fluid sample characteristics**	
Number of identified MII oocytes, n	136
Patients with FF samples from both ovaries, n	30
Patients with FF sample from one ovary, n	56
**Embryological outcome of the oocytes with matched FF**	
Fertilisation rate, n (%)	102/136(75.0)
TQ embryo on the 3rd day, n (%)	47/102(46.1)
TQ blastocyst on the 5th day, n (%)	13/42(30.95)

BMI, body mass index; FSH, follicle stimulating hormone; LH, luteinizing hormone; E2, estradiol; T, testosterone; P: progesterone; PRL, prolactin. Data are expressed as mean ± SEM.

### FF steroids and gonadotropic hormones and embryological data in patients

As seen in **Figure 2** and **Table 2**, the follicular fluid levels of LH and FSH were significantly higher in oocytes developing TQ blastocysts on the fifth day of culture compared with unfertilized oocytes (9.44 ± 2.32 vs. 5.30 ± 0.84mIU/ml, 9.32 ± 1.01 vs. 6.91 ± 0.62mIU/ml) (**Figures 2E, F**). Furthermore, the follicular fluid levels of 17-hydroxy pregnenolone (17-OHP), testosterone and cortisone level were lower in oocytes of TQ blastocysts on the fifth day than the unfertilized group (993.15± 69.65 vs. 1316.57 ± 97.90ng/ml; 0.49± 0.21 vs. 1.69 ± 0.52ng/ml, 15.37± 0.87 vs. 12.20 ± 0.82ng/ml) (**Figures 2G–I**). FF DHT concentrations were significantly lower in fertilized follicles after ICSI than in follicles with unfertilized oocytes (0.32 ± 0.02 vs. 0.45 ± 0.03ng/ml)(**Figure 2J**).

**Table 2:** Distribution of steroid and gonadotropic hormone concentrations in FF in relation to the corresponding oocytes: fertilisation and subsequent embryo development.

	No fertilisation after IVF (n=34)	Fertilisation after IVF (n=102)	TQ embryo on the 3^rd^ day (n=47)	TQ blastocyst on the 5^th^ day (n=13)
AMH (mIU/ml)	2.96 ± 0.34	2.91 ± 0.25	2.80 ± 0.37	2.89 ± 0.32
LH (mIU/ml)	5.30 ± 0.84	7.51 ± 0.76	7.66 ± 1.30	9.44 ± 2.32*
FSH (mIU/ml)	6.91 ± 0.62	8.34 ± 0.42	8.36 ± 0.56	9.32 ± 1.01*
PRL(mIU/l)	1173.15 ± 112.70	1089.60 ± 66.29	1277.82 ± 137.72	1109.46 ± 149.47
hCG(mIU/l)	36.03 ± 14.10	25.56 ± 2.04	24.11 ± 2.97	27.71 ± 6.80
Pregnenolone(ng/ml)	176.92 ± 20.56	214.01 ± 13.14	233.06 ± 20.18	200.41 ± 27.09
Progesterone(ng/ml)	8108.99 ± 534.01	8621.52 ± 330.46	8360.66 ± 570.05	7496.51 ± 469.58
17-OH Pregnenolone(ng/ml)	69.37 ± 3.46	66.83 ± 2.00	67.18 ± 2.76	60.16 ± 3.53
17-OHP(ng/ml)	1316.57 ± 97.90	1257.82 ± 53.22	1227.39 ± 74.45	993.15 ± 69.65*
21-OHP(ng/ml)	13.75 ± 0.36	15.47 ± 0.44	15.20 ± 0.76	13.42 ± 0.54
DHEA(ng/ml)	24.74 ± 1.69	27.22 ± 1.66	24.60 ± 2.04	22.52 ± 3.24
Testosterone(ng/ml)	1.69 ± 0.52	0.76 ± 0.16^#^	0.96 ± 0.34	0.49 ± 0.21*
A4(ng/ml)	7.20 ± 1.13	5.96 ± 0.65	6.10 ± 1.11	5.94 ± 1.47
DHT(ng/ml)	0.45 ± 0.03	0.32 ± 0.02^#^	0.32 ± 0.04^	0.32 ± 0.06*
Estriol(ng/ml)	8.26 ± 0.67	9.09 ± 1.13	9.75 ± 2.83	7.31 ± 0.87
Estrone(ng/ml)	84.56 ± 12.09	90.07 ± 10.29	71.57 ± 10.19	74.49 ± 22.12
Estradiol(ng/ml)	624.52 ± 52.06	585.18 ± 29.41	545.50 ± 42.00	493.84 ± 58.56
Corticosterone(ng/ml)	2.51 ± 0.32	2.50 ± 0.15	2.66 ± 0.30	3.36 ± 0.69
Cortisone(ng/ml)	15.37 ± 0.87	14.41 ± 0.59	14.56 ± 1.03	12.20 ± 0.82*
ALD(ng/ml)	0.058 ± 0.004	0.07 ± 0.004	0.07 ± 0.01	0.059 ± 0.01
Cortisol(ng/ml)	53.11 ± 2.89	51.16 ± 1.43	53.93 ± 2.52	60.04 ± 5.92
11-deoxycortisol (ng/ml)	2.15 ± 0.19	2.05 ± 0.17	2.05 ± 0.33	1.61 ± 0.22

AMH, anti-Müllerian hormone; hCG, human chorionic gonadotropin; 17-OHP: 17-hydroxy pregnenolone; 21-OHP, 21-hydroxy pregnenolone; DHT, Dihydrotestosterone; DHEA, dehydroepiandrosterone; A4, Androstenedione. Data are expressed as mean ± SEM. Differences between groups were considered significant for p ≤ 0.05.

#Student t-test p < 0.05 for no fertilisation vs. fertilisation;

^ Student t-test p < 0.05 for no fertilisation vs. TQ embryo on the 3rd day.

^∗^Student t-test p < 0.05 for no fertilisation vs. TQ blastocyst on the 5th day.

### Correlation of follicular fluid FSH and LH levels with embryo quality on day 5

**Figure 3:**
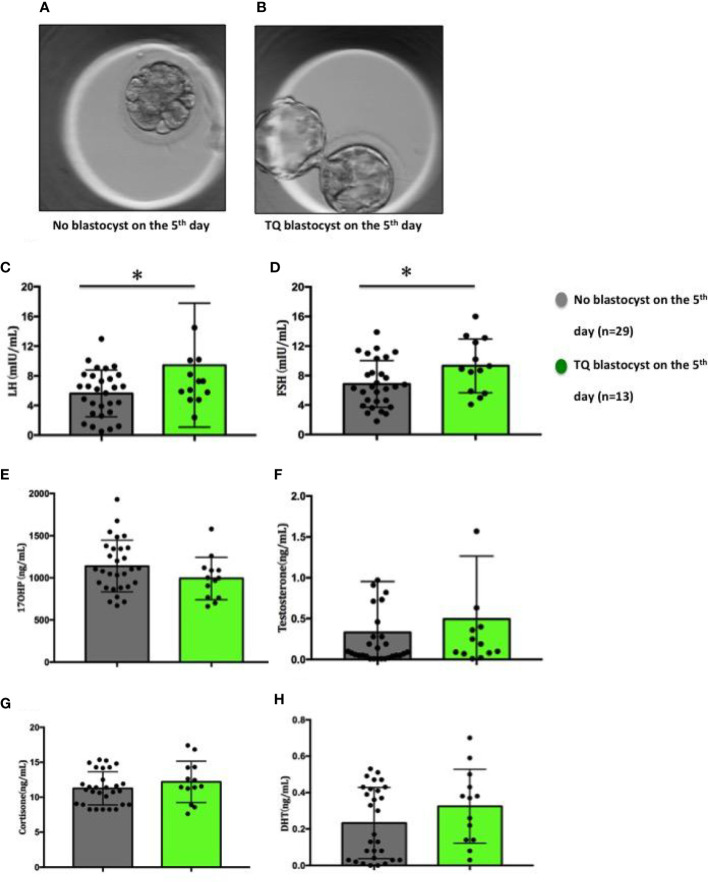
Figures of embryos and distribution of steroid and gonadotropic hormone concentrations in follicular fluid in relation to the embryo development on the 5^th^ day. **(A)** No blastocyst on the 5^th^ day; **(B)** TQ blastocyst on the 5^th^ day; **(C)** LH; **(D)** FSH; **(E)** 17-OHP; **(F)** Testosterone; **(G)** Cortisone; **(H)** Dihydrotestosterone. Comparison between groups was performed using Mann-Whitney U test. *p < 0.05.

To evaluate the possibility of using key reproductive hormones as potential indicators of oocytes developmental potential, we further divided the collected FF sample on the fifth day into two group: (1) Follicular fluid derived from oocytes fertilised but failed to reach blastocyst stage on the 5th day (**Figure 3A**, n=29); (2) Follicular fluid derived from oocytes fertilised and developed into a blastocyst on the 5th day (**Figure 3B**, n=13). Similarly, FF LH and FSH levels were significantly higher (LH: p=0.037; FSH: p=0.033) in the group 2 (LH: 9.45 ± 2.32mIU/ml; FSH: 9.32± 1.01mIU/ml) compared to the group 1 (LH: 5.63 ± 0.59mg/ml; FSH: 6.86 ± 0.59mIU/ml) (**Figures 3C, D**). There were no differences between the two groups in 17-OHP, testosterone, cortisone and DHT levels (**Figures 3E–H**).

### Correlation of follicular fluid hormone levels with embryo quality

**Figure 4:**
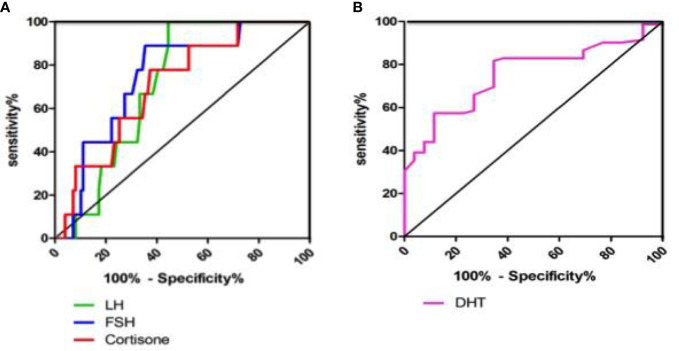
Receiver operating characteristic curve analysis of LH, FSH, Cortisone and DHT levels in FF. **(A)** Receiver operating characteristic curve analysis of LH, FSH and Cortisone levels in FF for top quality embryos on the 5th day from 136 matched embryos; **(B)** Receiver operating characteristic curve analysis of DHT level in FF for fertilisated embryos from 136 matched embryos.

The ROC analysis showed that FF LH levels predicted the TQ embryos on the fifth day with high sensitivity (100%) and specificity (55.6%), while FF FSH predicted the TQ embryos on the fifth day with 88.9% sensitivity and 64.6% specificity. Our results showed that 100% of the oocytes that matured in FF with LH levels≥5.75ng/ml led to develop TQ blastocysts on the fifth day of culture, while 88.9% of the oocytes with FF FSH level≥8.44ng/ml yielded the blastocysts on the fifth day of culture. The AUC for FF LH had a good value of 0.711, and that for FF FSH had a value of 0.744, implying that both are strong predictors of the TQ embryos on the fifth day (**Table 3**; **Figure 4A**).

**Table 3:** Receiver operating characteristic curve analysis of LH, FSH and Cortisone levels in FF for top quality blastocysts on the 5th day from 136 matched embryos.

TQ blastocyst on the 5th day	Cut-off value	AUC	Sensitivity	Specificity	SE	95% CI		P
LH	5.75	0.711	1	0.556	0.055	0.602	0.820	0.037
FSH	8.44	0.747	0.889	0.646	0.071	0.608	0.887	0.014
Cortisone	12.659	0.707	0.778	0.626	0.078	0.555	0.859	0.04

AUC,area under the ROC curve; SE, standard error; 95% CI, 95% confidence interval.

Furthermore, the cortisone level (AUC=0.707) also had certain significance as a predictor of blastocyst stage on the fifth day (**Figure 4A**). Cortisone had high sensitivity and specificity for predicting blastocyst, with a cutoff value of 12.66ng/ml. The ROC analysis of DHT showed that FF DHT level predicted the fertilization embryos with high sensitivity (81.7%) and specificity (65.4%), indicating that DHT is a predictor of fertilized embryos (**Table 4**; **Figure 4B**). 81.7% of the oocytes with FF DHT level ≤0.432ng/ml led to fertilized (**Table 4**).

**Table 4:** Receiver operating characteristic curve analysis of DHT levels in FF for fertilised oocytes from 136 matched oocytes.

Fertilisation after ICSI	Cut-off value	AUC	Sensitivity	Specificity	SE	95% CI		P
DHT	0.432	0.756	0.817	0.654	0.049	0.66	0.851	<0.01

AUC,area under the ROC curve; SE, standard error; 95% CI, 95% confidence interval.

### Mitochondrial ETC mRNA expression in FF exosomes

**Figure 5:**
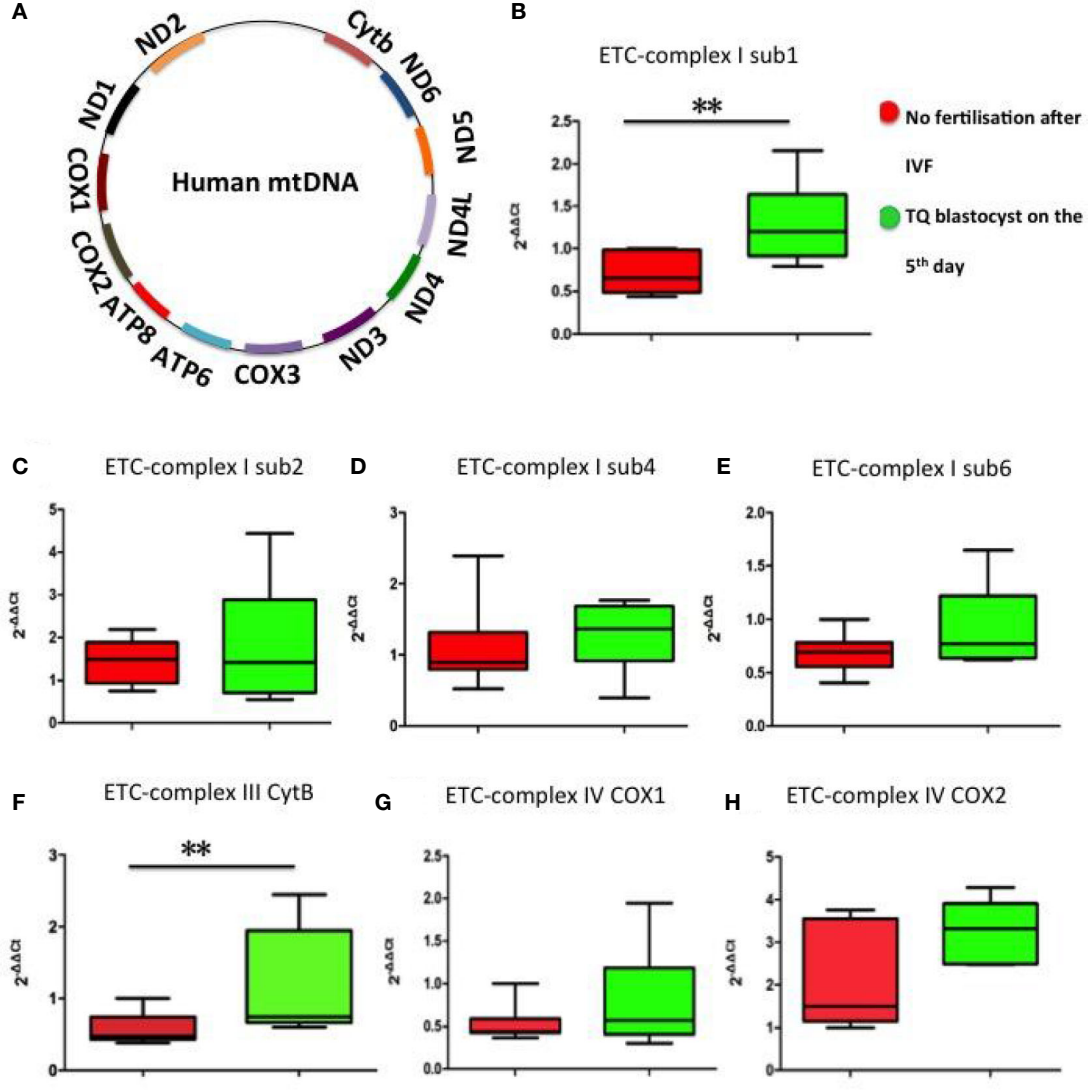
Representation of the mitochondrial ETC genes and mRNA expression levels of mitochondrial ETC genes in FF exosomes. **(A)** Representation of the 13 mitochondrial ETC genes. **(B)** ETC complex I sub1; **(C)** ETC complex I sub2; **(D)** ETC complex I sub4; **(E)** ETC complex I sub6; **(F)** ETC complex III CytB; **(G)** ETC complex IV COX1; **(H)** ETC complex IV COX2. Comparison between groups was performed using t-test. Data are presented as mean ± SD; **p< 0.01.

Mammalian mitochondrial DNA (mtDNA) consists of a 16.5kb double-stranded circular DNA molecule. All 13 polypeptide genes in mtDNA are involved in the production of mitochondrial complex components. MtDNA encodes seven of the 43 subunits of complex I (ND1, 2, 3, 4, 4L, 5, and 6), one of the 11 subunits of complex III (cytochrome b), three of the 13 subunits of complex IV (COX1, COX2 and COX3) and twoof 17 subunits of complex V (ATPase 6 and ATPase 8) (24) (**Figure 5A**). The mRNA expression levels of mitochondrial ETC genes from the FF exosomes were examined in our study, only ETC complex I sub1, ETC complex I sub2, ETC complex I sub4, ETC complex I sub6, ETC complex III CytB, ETC complex IV COX1 and ETC complex IV COX2 can be detected in FF exosomes. The results showed significantly increased ETC complex I sub1 (**Figure 5B**) and ETC complex III CytB (**Figure 5F**) mRNA expression in the group of oocyte fertilized and developed into a TQ blastocyst as on day 5 than the group of no fertilisation, whereas the other ETC complex mRNA expression were not affected (p> 0.05) (**Figures 5C, D, E, G, H**).

**Figure 6:**
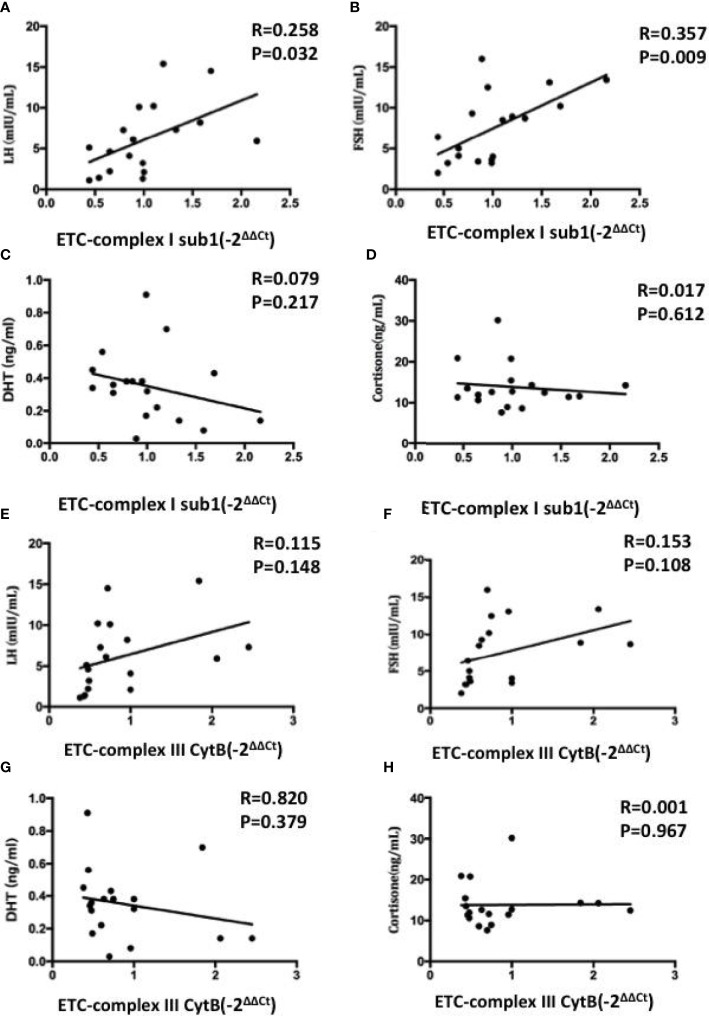
Correlations between the mRNA expression levels of mitochondrial ETC genes in FF exosomes with LH **(A, E)**, FSH **(B, F)**, DHT **(C, G)** and cortisone **(D, H)** levels. Pearson test was used for normally distributed variables, and Spearmen test was used for non-normally distributed parameters. Values are significant at p < 0.05.

We further elucidated whether mRNA expression levels of mitochondrial ETC genes can predict the hormone in FF. Pearson correlation analysis showed that ETC complex I sub1 mRNA level was positively and significantly correlated with LH and FSH levels (**Figures 6A, B**), whereas the other ETC complex mRNA expression were not correlated obviously (p> 0.05) (**Figures 6C–H**). The increases in the ETC complex I sub1 expression levels of were accompanied by the changes in hormonal levels in FF. The lower ETC expression indicated the impaired mitochondria function observed in oocytes, which correlates with the ability of the human oocytes to be fertilized.

## Discussion

Because the oocyte microenvironment is essential for healthy oocyte development and maturation, biochemical analysis of follicular fluid components can provide biomarkers of oocyte health and maturation. Most human oocytes extracted during *in vitro* fertilization (IVF) do not develop into viable blastocysts (25, 26). During oogenesis, understanding the developmental capacity of the oocyte, also known as oocyte quality, is a major factor in improving reproductive success rate.

In the present study, FF matched to unfertilized oocytes after ICSI had the lowest LH and FSH levels. Compared with the no blastocyst embryos and blastocyst embryos, LH and FSH were also lower in the no blastocyst group. Follicle-stimulating hormone and luteinizing hormone are hormones produced by the pituitary gland and are essential for promoting follicle growth, differentiation and maturation (27). LH binds the G protein–coupled receptors in mural granulosa cells and activates the cAMP system. FSH binds its GPR receptor (FSHR), at the same time, collaborating with estrogen/ER signal pathway. S Marchiani et al. (28) found LH supplementation in women with a low response can improve ovarian steroid production, better mimicking physiological production, and may contribute to improved ovarian response. With the addition of FSH, LH or HCG (29) to the IVM medium, immature oocytes rapidly undergo spontaneous oocyte meiotic maturation. Oocyte maturation begins with the transformation of germinal vesicle (GV) oocytes into MI oocytes and then into MII oocytes. LH initiates oocyte meiotic maturation (30). Funahashi et al. (31) exposed porcine oocytes to the cAMP analogue dbcAMP. Although the rate of oocyte maturation did not increase, there was an increase in oocyte quality. The blastocyst rate in the treated group was higher than that in the untreated group (21.5% vs 9%). Our study indicated that FF-LH and FF-FSH levels are good predictors of embryo quality on the fifth day and the levels of LH and FSH are rise with the development of oocytes, which was consistent with previous research results. However, previous study (32) also found the excessive FSH of follicular fluid could interfere with oocyte meiosis, resulting in the formation of aneuploid gametes.

In our present study, cortisone level in FF is another predictor of embryo quality on the fifth day, A E Michael et al. (33) found concentrations of cortisone in FF samples obtained from IVF-ET conceived patients was significantly lower than that obtained from non-conception cycles. S D Keay et al. (34) showed the higher cortisol: cortisone ratios in conception cycles may be important for eventual oocyte maturation and embryo implantation. Sabina Lewicka et al. (35) found lower follicular cortisone and higher cortisol/cortisone ratio in both serum and FF are associate with the high clinical pregnancy. In our present study, cortisol: cortisone ratios also higher in the TQ blastocyst on the fifth day than the no fertilization group.

11β-hydroxysteroid dehydrogenase (11β-HSD) catalyzes the interconversion of cortisol (an active glucocorticoid) and cortisone (an inactive steroid). The interconversion of cortisol to cortisone is determined by two 11β-HSD isoforms’ expression. Type 1 depends on NADPH and mainly acts as a reductase to produce cortisol from cortisone, while type 2 depends on NAD+ dehydrogenase to convert cortisol to cortisone (36). 11β-HSD is differentially regulated in human granulosa cells. Type 2 11β-HSD is expressed during the follicular phase (cortisol to cortisone conversion). Following the onset of the LH, type1 11β-HSD being expressed (37) which favours cortisol generation. Follicular cortisone decreases immediately before ovulation in women, suggesting that steroids may play a physiological role in oocyte maturation and ovulation (38). All these results indicated cortisone level could predict the embryo quality to some extent.

In our study, FF DHT level predicted unsuccessful fertilization with 81.7% sensitivity and 65.4% specificity. DHT, as one of the androgen, plays important roles in female reproduction in healthy and pathological states. Research on DHT in human embryos or follicular fluid is limited, and the existing research on DHT mainly focuses on mouse studies. Mouse study (39) has found long-term DHT treatment replicated a range of ovarian, endocrine, and metabolic features of human PCOS. Another research in mouse has the opposite conclusion that the high dose of DHT prompted normal follicular development in granulosa cells of the ovarian follicle (40). We are currently expanding the sample size to obtain more reliable results.

Electron transport chain (ETC) composed of complexes I-IV, the activity of which regulates mitochondrial oxidative phosphorylation (41). During follicular development, mitochondrial ATP is the main energy source for the FSH-dependent proliferation and differentiation of granulosa cells (42) and is produced by oxidative phosphorylation of mitochondria in ETC complexes (43). Our research showed significantly increased ETC complex I (**Figure 5B**) and ETC complex III CytB (**Figure 5F**) mRNA expression in the group of oocyte fertilised and developed into blastocyst on day 5. In addition, strong positive correlation was found between FF FSH and LH and ETC complex I mRNA expression, while no correlation between FF DHT and cortisone and ETC complex I/III mRNA expression. A large number of researches have proved mitochondrial dysfunction leads to a decline in oocyte quality, which affects embryonic development (44–46).

Follicles development depends mainly on surrounding granulosa cells for energy. FSH induced granulosa cell proliferation and differentiation, steroidogenesis, and LH receptor expression (47). LH then acts on receptors on granulosa cells in the preovulatory follicle, leading to oocyte maturation and ovulation (48). In our study, the TQ blastocysts group, with increased LH and FSH levels, induced granulosa cell proliferation and follicular fluid exosomes which are mainly released by granulosa cells. Oocyte and embryo development require optimal energy production, the increased mitochondrial ETC mRNA expression on the TQ blastocysts group indicated the sufficient source of ATP during preimplantation embryonic development. Both the steroid and mitochondrial function promoted granulosa cell proliferation and lead to the oocyte and embryo development.

There are some limitations throughout the current study. First, we did not collect enough data on patient IVF outcomes or pregnancies. Evaluation of FF hormone levels and IVF outcomes may be more valuable than day 5 blastocyst conditions. We are collecting additional FF samples and these results validated in a larger cohort to assess the effect of steroid and gonadotropic hormone levels in FF on IVF outcomes. Another potential limitation is that women undergoing ovarian stimulation had different individualized stimulation regimens as determined by treating clinicians. Although we included FSH treatment as a covariable, future studies should control for morel details of individual stimulation regimens. Finally, we have to admit that the present sample size was limited and small, even though some of the results are conclusive.

Our study is the first to correlate the changes of hormone levels in follicular fluid with the expression levels of mitochondrial complexes. The extraction of exosomes is time-consuming and laborious. By detecting the levels of LH and FSH levels in follicular fluid, the expression level of mitochondrial complexes can be reflected, which can predetermine subsequent embryonic developmental competence to some extent.

## Conclusion

With the widespread use of assisted reproductive techniques, the effects of these techniques on oocyte quality and mitochondrial function have been extensively studied. Oocyte maturation, fertilization and embryonic development require optimal mitochondrial function. Mitochondrial coding subunits that regulate respiratory chain complexes may play a key role in the relevant mechanisms. Our study is a preliminary exploration of hormone levels in follicular fluid and exosomes mitochondrial expression. The levels of FF steroids and gonadotropic hormones from single follicle can predetermine subsequent embryo development to some extent. Furthermore, impaired exosome mitochondrial dysfunction is a potiential event that causes hormone change in embryo development. We still need verify these results in a larger sample size and more cytology and animal study to further prove these mechanisms.

## Data availability statement

The original contributions presented in the study are included in the article/**Supplementary Material**. Further inquiries can be directed to the corresponding authors.

## Ethics statement

The studies involving human participants were reviewed and approved by Zhongshan Hospital, Fudan University (Shanghai, China). The patients/participants provided their written informed consent to participate in this study.

## Author contributions

LY, ML and XD collected samples and clinical data; SL and XD performed assisted reproductive technology; ZW tested the steroid of follicular fluid by LC/MS; SX tested the hormone of follicular fluid by immunoassays; JZ, TL, BP, BW and WG designed the study; LY analyzed the data and wrote the paper. The authors read and approved the final manuscript.

## Funding

This work was supported by grants National Natural Science Foundation of China 81972000, 81772263, 82172348, Specialized Fund for the clinical researches of Zhongshan Hospital affiliated Fudan University 2018ZSLC05, Constructing project of clinical key disciplines in Shanghai shslczdzk03302, Shanghai Medical Key Specialty ZK2019B28, Key medical and health projects of Xiamen YDZX20193502000002 (to WG); National Nature Science Foundation of China 81902139 and Specialized Fund for the clinical researches of Zhongshan Hospital affiliated Fudan University 2020ZSLC54(to BW); National Natural Science Foundation of China 82202607 (to LY); National Natural Science Foundation of China 81971345 (to SL); National Natural Science Foundation of China 82001545 (to ML).

## Acknowledgments

The authors would like to thank the patients for their participation in this study.

## Conflict of interest

The authors declare that the research was conducted in the absence of any commercial or financial relationships that could be construed as a potential conflict of interest.

## Publisher’s note

All claims expressed in this article are solely those of the authors and do not necessarily represent those of their affiliated organizations, or those of the publisher, the editors and the reviewers. Any product that may be evaluated in this article, or claim that may be made by its manufacturer, is not guaranteed or endorsed by the publisher.

## Abbreviations

FF, Follicular fluid; LC-MS/MS, Liquid chromatography-tandem mass spectrometry; ETC, Electron transport chain; ART, Assisted reproductive technology; IVF, *In vitro* fertilization; hCG, Human chorionic gonadotropin; ICM, Inner cell mass; TE, Trophectoderm; EVs, Extracellular vesicles; qRT-PCR, Quantitative RT-PCR.
