# Supplementary material for: Follicular fluid steroid and gonadotropic hormone levels and mitochondrial function from exosomes predict embryonic development
Source: Front Endocrinol (Lausanne). 2022 Nov 9;13:1025523. doi: 10.3389/fendo.2022.1025523 (PMC9682035; doi:10.3389/fendo.2022.1025523)
Supplement: Supplementary file 1 [file Table_1.docx]

Supplemental material

Table S1: The primer sequences for RT-PCR

| **Gene** | **Sense primer (5'-3')** | **Antisense primers (5'-3')** |
| --- | --- | --- |
| ETC-CI sub1 | ATTACTTCTGCCAGCCTGACC | GGCCCGGTTTGTTTCTGCTA |
| ETC-CI sub2 | ATAAAACTAGGCCTCGCCCC | AGTCCTATGTGCAGTGGGAT |
| ETC-CI sub3 | TTGCATTCTGACTCCCCCAAAT | GCTTGTAGGGTCGAATCCGC |
| ETC-CI sub4 | TAATCGCACATGGCCTCACA | GCTGTGGATCCGTTCGTAGT |
| ETC-CI sub5 | ATGGTACGGACGAACAGACG | CGATGTCTCCGATGCGGTTA |
| ETC-CI sub6 | CCGCAAACAAAGATCACCCAG | GAAGGAGGGATTGGGGTAGC |
| ETC-CIII CytB | GGCTACGTCCTTCCATGAGG | TGGGATGGCTGATAGGAGGT |
| ETC-CIV COX1 | ATCACTACCAGTGCTAGCCG | CCTCCAGCGGGATCAAAGAA |
| ETC-CIV COX2 | CCGAGTCGTTCTGCCAATAGA | ACTGCTCATGAGTGGAGGAC |
| ETC-CIV COX3 | TGCAGGATTCTTCTGAGCGTT | AGGTCAGCAGCCTCCTAGAT |
| ETC-CV ATP6 | TCCCAATCGTTGTAGCCATCA | AGACGGTTGTTGATTAGGCGT |
| ETC-CV ATP8 | AACATTCCCACTGGCACCTT | TCGTTCATTTTAATTCTCAAGGGGT |
| Human 18S rRNA | CAGCCACCCGAGATTGAGCA | TAGTAGCGACGGGCGGTGTG |
